# A First-Principles Modeling of the Elastic Properties and Generalized Stacking Fault Energy of Ir-W Solid Solution Alloys

**DOI:** 10.3390/ma18153629

**Published:** 2025-08-01

**Authors:** Pengwei Shi, Jianbo Ma, Fenggang Bian, Guolu Li

**Affiliations:** 1 School of Materials Science and Engineering, Hebei University of Technology, Tianjin 300401, China; shipengwei2022@163.com; 2 Shanghai Advanced Research Institute, Chinese Academy of Sciences, Shanghai 201204, China; bianfg@sari.ac.cn

**Keywords:** iridium–tungsten alloys, first-principles simulation, elastic properties, generalized stacking fault energy, toughness improvement

## Abstract

Iridium, with its excellent high-temperature chemical inertness, is a preferred cladding material for radioisotope batteries. However, its inherent room-temperature brittleness severely restricts its application. In this research, pure Ir and six Ir-W solid solutions (Ir_31_W_1_ to Ir_26_W_6_) were modeled. The effects of W on the elastic properties, generalized stacking fault energy, and bonding properties of Ir solid solution alloys were investigated by first-principles simulation, aiming to find a way to overcome the intrinsic brittleness of Ir. With the W concentration increasing from 0 to 18.75 at %, the calculated Cauchy pressure (*C*_12_–*C*_44_) increases from −22 to 5 GPa, Pugh’s ratio (*B*/*G*) increases from 1.60 to 1.72, the intrinsic stacking fault energy reduces from 337.80 to 21.16 mJ/m^2^, and the unstable stacking fault energy reduces from 636.90 to 547.39 mJ/m^2^. According to these results, it is predicted that the addition of W improves the toughness of iridium alloys. The alloying of W weakens the covalency properties of the Ir-Ir bond (the ICOHP value increases from −0.8512 to −0.7923 eV). These phenomena result in a decrease in the energy barrier for grain slip.

## 1. Introduction

Iridium (Ir) exhibits a high melting point (2443 °C), excellent high-temperature chemical inertness [1], and good compatibility with PuO_2_ fuel and external graphitic components at about 1400 °C. Consequently, Ir alloys provide an optimal series of cladding materials for radioisotope batteries [2]. The nuclear fuel cladding of radioisotope thermoelectric generators (RTGs) is also expected to have good strength and toughness properties. This is to prevent nuclear fuel from leaking in the case of an impact with the Earth in the event of a launch failure [3]. However, Ir, unlike other face-centered cubic (FCC) metals (e.g., Cu, Ag, Au, etc.), exhibits inherent brittleness at room temperature (RT) [4]. It is reported that brittle transgranular fracture is the sole fracture mode of high-purity Ir at RT [4]. Research on the inherent brittleness of Ir and strategies to improve its toughness is a longstanding issue.

Doping and alloying efforts have been carried out to improve its mechanical strength and toughness [5,6,7,8]. It is reported that trace doping of Th and Ce (<30 ppm) significantly enhances the fracture toughness of Ir alloys [9]. This fact is closely related to the nanoscale ThIr_5_ and CeIr_5_ precipitates at grain boundaries. As early as the 1980s, the Oak Ridge National Laboratory in the United States had already developed DOP-26 (Ir-0.3W-0.006Th-0.005Al, wt%) with excellent mechanical properties and ductility [9,10]. DOP-26 has since become the cladding material of choice for NASA [11].

Theoretical calculations [12,13] suggested that the solid solutions of some elements (3.125 at %), such as Th, La, Ce, and Y, can significantly improve the toughness of Ir. However, the solubility of Th, La, Ce, and Y in Ir is extremely low. For example, the solubility of Th in Ir is about 30 ppm [10]. Though supersaturated solid solutions can be obtained with the rapid quenching technique, it is nearly impossible to improve their solubility in FCC Ir with traditional casting methods. Density functional theory (DFT) calculations suggest that W and Pt may improve the brittleness of Ir [12,13]. The early literature reported that the ductility of Ir alloys was slightly improved by alloying with 0.3% W [10]. Several experimental studies [14,15,16] on Ir-W-Al alloys suggested that alloying with W and Al can improve the high-temperature mechanical properties of iridium alloys. W is likely to be a preferred alloying element to improve the toughness of Ir. Improving the ductility of refractory high-entropy alloys (HEAs) is also a research hotspot because of their high strength and obvious brittleness [17]. It is reported that solid solution alloying [18], second-phase dissolution [19], compositional gradient design [20], valence electron concentration [21], and microstructure design can improve the toughness of HEAs. The primary purpose of this work is to investigate the effect of W solid solution alloying on the mechanical toughness of Ir-based alloys through DFT calculations. Hu et al. [22] predicted that the addition of V shortens the pseudo-energy gap and enhances the interaction force between Mo and W atoms in a NbMoTaW HEA by first-principles calculation. The addition of V improves the mechanical properties of the NbMoTaW HEA but does not improve the toughness.

The reported Pugh’s ratio (*B*/*G*) and Poisson’s ratio (ν) of pure Ir, with inherent brittleness, are about 1.61 and 0.24, respectively [23]. Elastic properties are closely related to the toughness of metal materials. When Pugh’s ratio is greater than 1.75 or Poisson’s ratio is greater than 0.26, the material is considered tough [24]. If they are below these values, the material is considered brittle.

The generalized stacking fault energy (GSFE), originally proposed by Vitek [25], is closely related to the slip and dislocation behavior. FCC metals generally exhibit high ductility, attributed to their close-packed atomic arrangement and 12 slip systems. The intrinsic stacking fault energy (γ_isf_) of Ir (about 420 mJ/m^2^ [26]) is much higher than that of Cu (about 51 mJ/m^2^), Ag (about 21 mJ/m^2^ [27], and Au (about 33 mJ/m^2^ [26]), which have good ductility. These values are closely related to the deformation and plane sliding nature of those metals. Pugh’s ratio (*B*/*G*) and stacking fault energy (SFE) are always employed to predict or estimate the toughness evolution of a material after alloying [28,29,30]. Shang et al. [28] reported that alloying elements in diluted Ni-based alloys and increasing the temperature induce a decrease in the SFE, which is linked to easier shear deformation. Tian et al. [29] reported that alloying elements such as Cr and W in FCC Co-based binary alloys can tune the SFE to modulate dislocation behavior. A lower SFE facilitates the dissociation of full dislocations into Shockley partials. This dissociation hinders cross-slips, reduces creep rates, and thus contributes to enhanced ductility.

The Ir-W equilibrium phase diagram shows that W exhibits high solubility in Ir (~19 at.% above 1700 °C, 17.8 at.% at 1400 °C) [31]. Alloying with W may significantly improve the toughness of Ir without reducing the alloy strength. However, the effects of W on the elastic properties, stacking fault energy, and the bonding characteristics are still not exclusively reported. In the present work, Ir-W solid solutions (Ir_31_W_1_, Ir_30_W_2_, Ir_29_W_3_, Ir_28_W_4_, Ir_27_W_5_, and Ir_26_W_6_) cubic atomic models and eight-layer ABCABCAB stacking models were constructed. Their equilibrium lattice constants, formation energies, elastic properties, electronic structures, and GSFE were calculated using first-principles calculations. For the Ir-W solid solutions, the effects of W on lattice distortion and electronic charge distribution within the Ir matrix were analyzed. The elastic properties of three intermetallic compounds (Ir_4_W, Ir_3_W, and IrW) were also investigated for comparison. The evolution of Cauchy pressure (*C*_12_−*C*_44_), Pugh’s ratio (*B*/*G*), GSFE, and bonding characteristics was discussed to predict the evolution of toughness. These findings will improve the understanding of why W enhances the toughness of Ir-based alloys.

## 2. Methodology

First-principles calculations were performed using the Vienna ab initio simulation package [32,33] (VASP) based on density functional theory [34] (DFT). The generalized gradient approximation (GGA) with the Perdew–Burke–Ernzerhof [35] (PBE) functional was employed to describe the electron–ion interaction. The electron configurations for Ir and W atoms are [Xe] 4f^14^5d^7^6s^2^ and [Xe] 4f^14^5d^4^6s^2^, respectively. The maximum plane-wave basis cutoff energy was 550 eV. The convergence criteria for the electronic and ionic steps were set to 1 × 10^−6^ eV/atom and 0.005 eV/Å. The k-point mesh was divided into 8 × 8 × 8 by the Monkhorst–Pack method for pure Ir and Ir-W solid solutions. The electronic density of states (DOS) was calculated using the linear tetrahedron method with Blöchl correction. All the calculations were carried out assuming a non-magnetic state. The crystal orbital Hamilton population (COHP) analysis was performed using the Local Orbital Basis Suite Towards Electronic-Structure Reconstruction (LOBSTER) procedure [36]. COHP partitions the electron density into bonding, non-bonding, and antibonding sections. Negative COHP values indicate bonding states, while positive COHP values indicate antibonding states [37,38,39,40].

**Figure 1:**
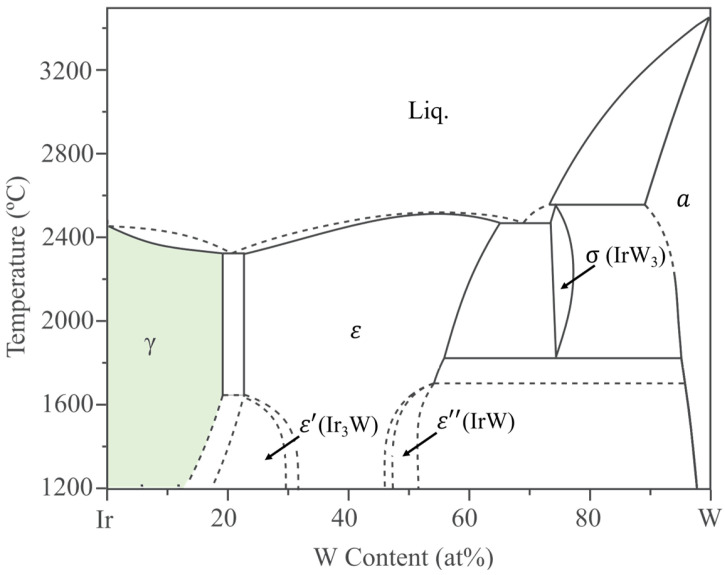
Phase diagram of Ir-W binary alloy [15,31].

The present research focuses on polycrystalline Ir-W alloys by casting. According to the Ir-W binary phase diagram [31] (Figure 1), the γ-Ir solid solution (with space group Fm$\bar{3}$m) has a solubility limit of approximately19 at.% W above 1700 °C.

Pure Ir and six Ir-W solid solutions (Ir_31_W_1_, Ir_30_W_2_, Ir_29_W_3_, Ir_28_W_4_, Ir_27_W_5_, and Ir_26_W_6_) with 2 ×  2 × 2 supercell models containing 32 atoms were constructed in this work. Within those solid solution supercells, Ir atoms were randomly replaced by W atoms. The special quasi-random structure (SQS) was established using the Alloy Theory Automation Toolkit [41,42,43] (ATAT) code. SQS simulates the random state of solute alloys by constructing supercells with special atomic positions that minimize the difference between their atomic correlation functions and that of the absolute random crystal cells. It is possible that the W is not uniformly distributed in a real alloy. There may be W-rich regions or W-regions in a real Ir-W alloy. The non-uniform distribution of W atoms in a real solid solution alloy was not addressed in the present research.

**Figure 2:**
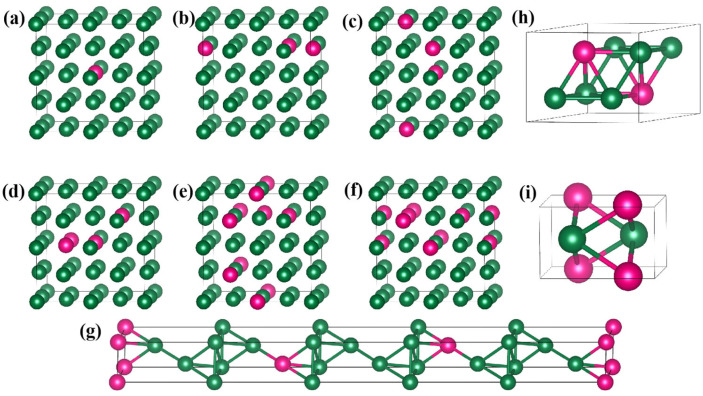
Structures of Ir-W alloys: (**a**) Ir_31_W_1_; (**b**) Ir_30_W_2_; (**c**) Ir_29_W_3_; (**d**) Ir_28_W_4_; (**e**) Ir_27_W_5_; (**f**) Ir_26_W_6_; (**g**) Ir_4_W; (**h**) Ir_3_W; (**i**) IrW. Green and red spheres denote Ir and W atoms, respectively.

According to the Ir-W binary phase diagram [31] (Figure 1), there are high temperature phases (ε) with W content of 22~66 at.%, stable-phase Ir_3_W (ε′, with space group P6_3_/mmc) and IrW (ε′′, with space group Pmma) on the Ir-rich side, and high-temperature-phase IrW_3_ (σ, with space group P4_2_/mnm) on the W-rich side. Diffusion coupling experiments with EPMA confirmed there are γ, ε′, ε, and ε′′ phases on the Ir-rich side with an increase in W content at 1500 °C [15]. Above 1650 °C, there are γ phases and ε phases on the Ir-rich side, with a two-phase region (γ + ε) located at approximately 19~22 at % W. The structure of Ir_4_W (with space group R$\bar{3}$m) was reported by the Materials Project database (mp-1223666) [44]; however, experimental studies on Ir_4_W are not reported. The 1×1×1 supercells of Ir_4_W, Ir_3_W, and IrW used in this work were derived from the Materials Project database [44]. For Ir_4_W, Ir_3_W, and IrW phases, the Brillouin zone was sampled with k-point meshes of 24 × 24 × 1, 12 × 11 × 11, and 11 × 18 × 10, respectively, generated by the Monkhorst–Pack scheme for geometry optimization and mechanical properties calculations. The high-temperature phases ε and σ (IrW_3_) were not considered in this work. The details of the Ir-W alloy supercells are illustrated in Figure 2. The key DFT calculation parameters used in the modeling are compared in Table 1.

### 2.1. Calculation of Lattice Constant and Supercell Length

The Birch–Murnaghan equation of state (EOS) [45] was employed to fit the pressure–volume data points for pure Ir and Ir-W solid solutions to determine their equilibrium volumes. The EOS is expressed as follows:

(1) $$P\left(V\right)=\left(\frac{B_{0}}{B_{0}^{\prime }}\right)\left\{{\left(\frac{V_{0}}{V}\right)}^{B_{0}}-1\right\}$$

where $P\left(V\right)$, V, ${V_{0},B}_{0},andB_{0}^{\prime }$ are the external pressure, volume, equilibrium volume, bulk modulus, and first derivative of bulk modulus to pressure, respectively. The zero-pressure volume *V*_0_ is defined as the equilibrium volume. The supercell length is calculated from $\sqrt[{3}]{V_{0}}$. The lattice constant of pure Ir is half its supercell length. The lattice constants of Ir_4_W, Ir_3_W, and IrW were obtained by the structural relaxation method in VASP with the GGA-PBE functional. 

### 2.2. Calculation of Formation Energy

The formation energy of Ir-W alloy, $\;\Delta E\left(Ir_{x}W_{y}\right)$, was calculated by the following equation [46]:

(2) $$\Delta E\left(Ir_{x}W_{y}\right)=\frac{E_{total}\left(Ir_{x}W_{y}\right)-{xE}_{atom}\left(Ir\right)-{yE}_{atom}\left(W\right)}{x+y}$$

where $E_{total}\left(Ir_{x}W_{y}\right)$ is total energy of the Ir-W alloy supercell. $E_{atom}\left(Ir\right)\;$ and $E_{atom}(W)$ are the energy of individual atoms of the FCC structure Ir and BCC structure W, respectively. *x* and *y* are the number of Ir and W atoms, respectively.

### 2.3. Calculation of Elastic Properties

The elastic properties were calculated by the stress–strain method [47]. The bulk modulus (*B*) and shear modulus (*G*) were obtained by the Voigt–Reuss–Hill approach [48]. For FCC structure alloys,

(3) $$B_{v}=B_{R}=\frac{C_{11}+2C_{12}}{3}$$

(4) $$G_{v}=\frac{C_{11}-C_{12}+3C_{44}}{5}$$

(5) $$G_{R}=\frac{5\left(C_{11}-C_{12}\right)C_{44}}{4C_{44}+3{(C}_{11}-C_{12})}$$

where $B_{v}$, $B_{R}$, $G_{v}$, and $G_{R}$ are the bulk and shear modulus from Voigt and Reuss models, respectively.

For the FCC system, the elastic constants follow the relationship *C*_11_ = *C*_22_ = *C*_33_, *C*_44_ = *C*_55_ = *C*_66_, *C*_12_ = *C*_23_ = *C*_13_, exhibiting three independent components, namely *C*_11_, *C*_12_, and *C*_44_. However, the addition of W, for example, in Ir_31_W_1_, Ir_30_W_2_, Ir_29_W_3_, Ir_28_W_4_, Ir_27_W_5_, and Ir_26_W_6_, slightly distorts the FCC symmetry of the supercell, resulting in minor differences between these parameters, e.g., *C*_11_, *C*_22_, and *C*_33_. The elastic constants were averaged according to Formulas (6)–(8), and the elastic moduli were subsequently calculated using Formulas (3)–(5).

(6) $$\bar{C_{11}}=\frac{1}{3}\left(C_{11}+C_{22}+C_{33}\right)$$

(7) $$\bar{C_{12}}=\frac{1}{3}\left(C_{12}+C_{23}+C_{13}\right)$$

(8) $$\bar{C_{44}}=\frac{1}{3}\left(C_{44}+C_{55}+C_{66}\right)$$

Formulas for calculating the elastic modulus of trigonal (Ir_4_W), hexagonal (Ir_3_W), and orthorhombic (IrW) structured alloys can be found in [49]. The bulk modulus (*B*) and shear modulus (*G*) were calculated using the Hill approximation, which is represented by the following formulas [48].

(9) $$B=\frac{B_{V}+B_{R}}{2}$$

(10) $$G=\frac{G_{V}+G_{R}}{2}$$

The Young’s modulus (E) and Poisson’s ratio (v) were derived from *B* and *G*.

(11) $$E=\frac{9BG}{3B+G}$$

(12) $$v=\frac{3B-2G}{6B+2G}$$

### 2.4. Calculation of Generalized Stacking Fault Energy

The specific formula for calculating GSFE is as follows [50]:

(13) $$\gamma (d)=\frac{E(d)-E_{0}}{A}$$

where E(d) is the energy of the crystal after deformation with a slip displacement *d*, *E*_0_ is the energy of the stacked configuration without slipping, and *A* is the area of the face defect region.

**Figure 3:**
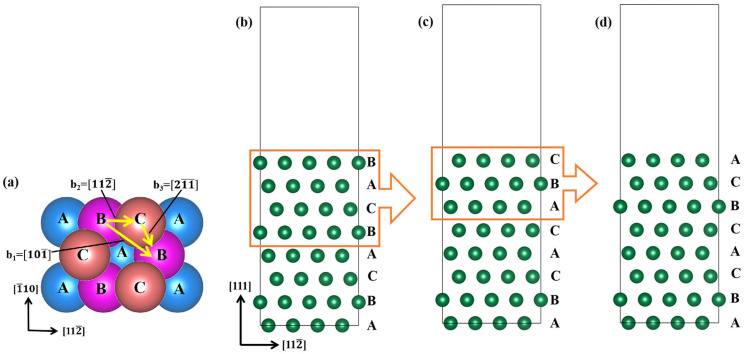
(**a**) Top view geometry of a (111) plane, where yellow arrows denote dislocations: full dislocation b_1_ and partial dislocations (b_2_, b_3_); (**b**) stacking model without slipping; (**c**) layered fault model after slipping 1.0b; (**d**) twinning fault model after further slipping 1.0b based on the layered fault model.

The close-packed (111) plane was employed as the slipping plane in this work. Supercell models with an 8-layer ABCABCAB stacking sequence, containing 128 atoms, were constructed by cutting along the [$\bar{1}\bar{1}0$] and [11$\bar{2}$] directions, with a 15 Å vacuum layer along the [111] direction. The supercell top view is shown in Figure 3a. In the FCC Ir structure, full dislocations b_1_ dissociate into partial dislocations (b_2_, b_3_). The slip path for the GSFE calculation was along the [11$\bar{2}$] direction, denoted as b_2_. The [$\bar{1}\bar{1}0$] direction view of the supercell without slipping is shown in Figure 3b.

Pure Ir and six Ir-W solid solutions (Ir_31_W_1_, Ir_30_W_2_, Ir_29_W_3_, Ir_28_W_4_, Ir_27_W_5_, and Ir_26_W_6_) with 2 × 2 × 2 supercell models containing 32 atoms were constructed in this work. For these solid solution supercells, Ir atoms were randomly replaced by W atoms. SQS simulates the random state of solute alloys by constructing supercells with special atomic positions, minimizing the difference between their atomic correlation functions and those of absolutely random crystal cells. It is possible that the W is not uniformly distributed in a real alloy. There may be W-rich regions or W-regions in a real Ir-W alloy. The non-uniform distribution of W atoms in a real solid solution alloy was not considered in the present research.

For the GSFE calculations, the total slip displacement (*b* = *a*/6 <11$\bar{2}$>) along the (111) plane was divided into ten equal increments of 0.1b each. The crystal energies E(d) at various slip displacements (0.2b, 0.4b, 0.5b, 0.6b, 0.8b, 1.0b, 1.2b, 1.4b, 1.5b, 1.6b, 1.8b, and 2.0b) were calculated, and the generalized stacking fault energies γ(d) of the supercell model in different configurations were derived using Formula (13). All atoms were allowed to relax only in the direction perpendicular to the slip surface for structural optimization after each slip. The maximum plane-wave basis cutoff energy was set to 450 eV for calculating GSFE. The convergence criteria for electronic and ionic steps were set to 1 × 10^−5^ eV/atom and 0.01 eV/Å, respectively. The k-point mesh was divided into 4 × 4 × 1 using the Monkhorst–Pack method.

## 3. Results

### 3.1. Lattice Constant and Supercell Length

The calculated lattice constants of pure Ir, Ir_4_W, Ir_3_W, and IrW and supercell lengths for Ir-W solid solution 2 × 2 × 2 supercells, along with previously reported computational and experimental values, are compared in Table 2.

The calculated lattice constant of pure Ir, with space group Fm$\bar{3}$m, is 3.873 Å, being in agreement with the reported experimental results of 3.819 Å [51] and 3.840 Å [52] and the computational values [12,13,23,53,54], see Table 2. The lattice parameters of Ir_4_W, with space group R$\bar{3}$m (a = 2.751 Å, c = 33.850 Å), are consistent with the reported values from the Materials Project database (www.materialsproject.org, ID: mp-1223666, accessed on 13 June 2024) [44]. The computed lattice constants of Ir_3_W (ε′), with space group P6_3_/mmc (a = 5.540 Å, c = 4.421 Å), and IrW (ε′′), with space group Pmma (a = 4.473 Å, b = 2.779 Å, c = 4.848 Å), are also in agreement with the reported computational [46] and experimental data [55]. The supercell length of an Ir 2 × 2 × 2 supercell (7.746 Å) is twice that of the lattice constant. The supercell length of Ir-W solid solution 2 × 2 × 2 supercells increases linearly with increasing W concentration. When the concentration of W reaches 18.75 at % (Ir_26_W_6_), the supercell length of Ir_26_W_6_ reaches 7.788 Å (see Table 2). These parameters reach a linear fit of *L* = 7.74461 + 0.22514*x*, with adjusted R^2^ = 0.99535. Here, *L* is the supercell length of Ir-W solid solution 2 × 2 × 2 supercells, *x* is the W concentration. The mean Ir-Ir bond lengths in Ir-W alloys increased with increasing W content (see Table 2). These phenomena may arise from the larger atomic radius of W (1.41 Å) compared to that of Ir (1.36 Å) [56]. These converged lattice constants and supercell lengths were used as initial geometric parameters for subsequent calculations, e.g., formation energy, elastic properties, stacking fault energy, and electronic structure.

### 3.2. Formation Energy

**Figure 4:**
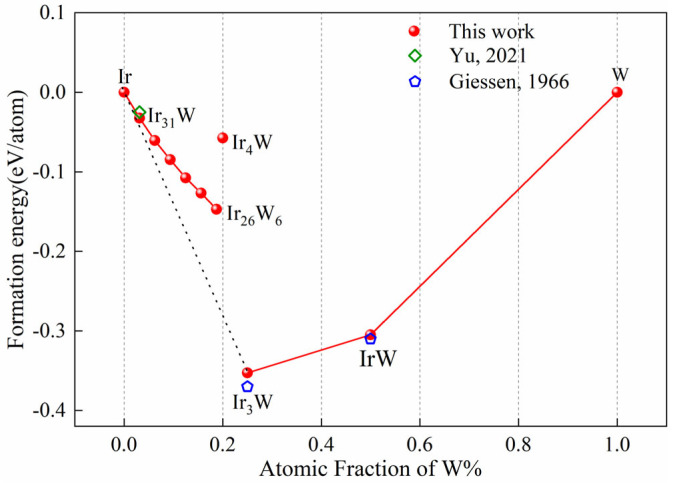
The formation energy of Ir_31_W_1_, Ir_30_W_2_, Ir_29_W_3_, Ir_28_W_4_, Ir_27_W_5_ and Ir_26_W_6_, Ir_4_W, Ir_3_W, and IrW [13,55].

The formation energies of the Ir-W solid solutions and Ir_4_W, Ir_3_W, and IrW compounds are shown in Figure 4. The calculated formation energies of Ir_3_W and IrW (−0.353 eV/atom and −0.305 eV/atom) are consistent with the results of previous studies [55]. The formation energies of the solid solutions and Ir_4_W (−0.058 eV/atom) are all negative, but above the dashed line connecting Ir and Ir_3_W, indicating that they are metastable relative to Ir_3_W at zero temperature. This fact indicates that Ir_3_W is likely to precipitate after appropriate heat treatment, even with low W concentrations. For example, according to the Ir-W phase diagram, when Ir_26_W_6_ is annealed at 1200 °C, the Ir_3_W phase will precipitate from Ir_26_W_6_ solid solution. It is strongly suggested that the IrW compound will not precipitate in the Ir-W solid solution. Formation energy of Ir_4_W (with space group R$\bar{3}$m) is much less negative than that of Ir_26_W_6_ and Ir_3_W. This finding does not support Ir_4_W being a stable phase. The structure of Ir_4_W (with space group R$\bar{3}$m) was obtained from the Materials Project database (mp-1223666) [44]. There are still no experimental reports about Ir_4_W. To find out whether it is a metastable phase, further research on the Ir_4_W phase is needed.

### 3.3. Elastic Properties

The elastic constants of Ir, as well as Ir-W alloys, are presented in Table 3 together with the previously reported calculated values for comparison.

**Figure 5:**
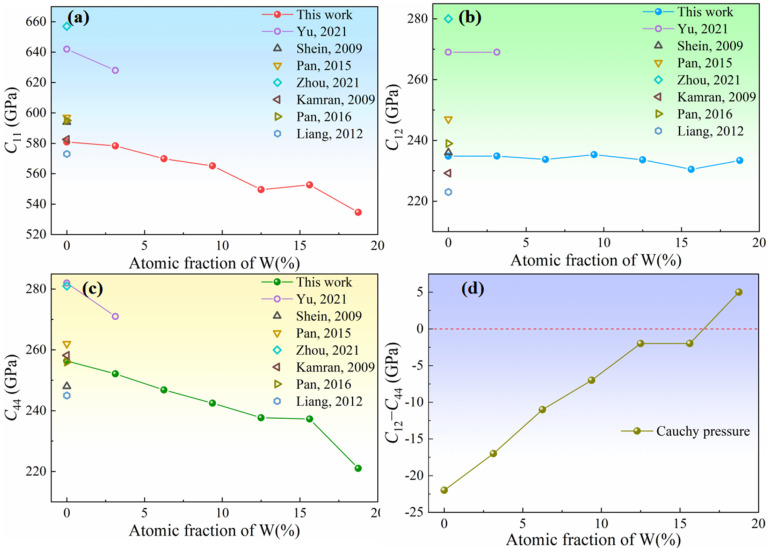
The elastic constants of Ir-W solid solution alloys (**a**) *C*_11_; (**b**) *C*_12_; (**c**) *C*_14_; and (**d**) *C*_12_−*C*_44_ and previously reported values [12,13,23,53,54,57,58].

The elastic constants *C*_11_ and *C*_44_ of Ir-W solid solution alloys decrease significantly with increasing W content, while *C*_12_ keeps nearly constant (see Figure 5a–c). These behaviors result in an increase of the Cauchy pressure (*C*_12_−*C*_44_), from −22 GPa to 5 GPa, as shown in Figure 5d. The Cauchy pressure has also been employed to discuss the ductility of materials by Pettifor [59]. A positive value of *C*_12_−*C*_44_ indicates good toughness, whereas a negative value indicates brittleness [60]. The increasing nature of *C*_12_−*C*_44_ with increasing W concentration suggests that the toughness of the Ir-W solid solutions is improved.

Table 4 presents the calculated elastic modulus, Pugh’s ratio (*B*/*G*), and Poisson’s ratio v of pure Ir and Ir-W alloys, together with the previously reported values for comparison. For the Ir-W solid solutions, the *B*/*G* ratio increases from 1.60 to 1.72 monotonously with increasing W concentration. ν increases from 0.241 to 0.257.

**Figure 6:**
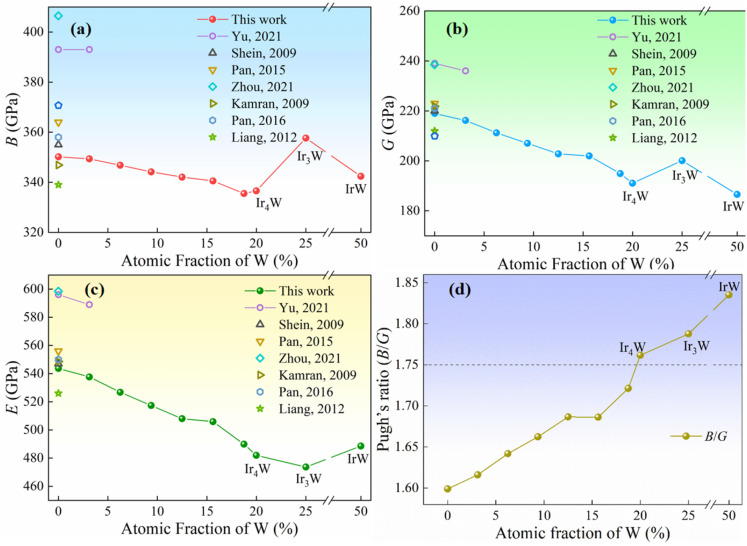
Elastic modulus parameters of Ir-W alloys, including (**a**) bulk modulus *B*, (**b**) shear modulus *G*, (**c**) Young’s modulus *E*, and (**d**) Pugh’s ratio (*B*/*G*) and previously reported values [12,13,23,53,54,57,58].

Figure 6a–c depict the elastic modulus (*B*, *G*, and *E*) of Ir-W alloy as a function of W content. The *B*, *G*, and *E* decrease with increasing W content within the Ir-W solid solution range. Figure 6d illustrates the evolution of the *B*/*G* ratio as a function of W content. The *B*/*G* ratio is commonly used to assess the toughness and brittleness of materials and exhibits a positive correlation with toughness [60,62,63]. As mentioned above, when the *B*/*G* ratio is greater than 1.75 or Poisson’s ratio is greater than 0.26, the material is considered tough [24,64] and vice versa. The increasing Cauchy pressure (*C*_12_−*C*_44_), *B*/*G* ratio, and Poisson’s ratio indicate that the incorporation of W atoms improves the toughness of Ir-W alloys.

The *B*/*G* ratios of intermetallics, i.e., Ir_4_W, Ir_3_W, and IrW, are larger than the critical value of 1.75 and larger than those of pure Ir and the Ir-W solid solutions. This fact indicates that these compounds may be tough and that they are tougher than pure Ir and Ir-W solid solutions. It is expected that the Ir_4_W or Ir_3_W phase will not deteriorate the toughness of the Ir alloy when they precipitate in the Ir-W solid solution alloy after appropriate heat treatment. The addition of W atoms is expected to weaken the Ir-Ir bond, leading to a decrease in elastic modulus. Further analysis of the electronic structure is stated in Section 3.5.

### 3.4. Generalized Stacking Fault Energy

**Figure 7:**
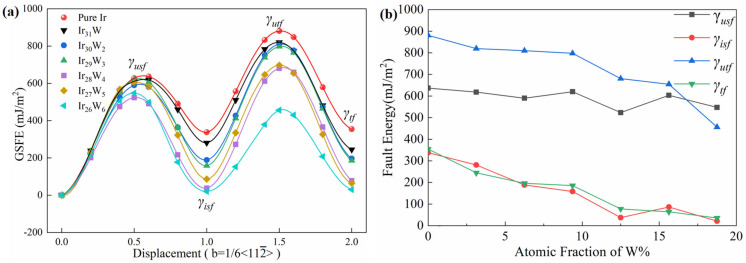
(**a**) GSFE curves of pure Ir and Ir-W solid solutions; (**b**) the evolution of intrinsic stacking fault energy (*γ_isf_*), unstable stacking fault energy (*γ_usf_*), twinning fault energy (*γ_tf_*), and unstable twinning fault energy (*γ_utf_*) as a function of W atomic fraction.

Figure 7a depicts the GSFE curves of pure Ir and Ir-W solid solutions. The GSFE curves characterize the energy variation during atomic displacement on the slip plane. The unstable SFE (*γ_usf_*) is at 0.5b, and the intrinsic SFE is at 1.0b. The former is always considered as the energy barrier for the formation of intrinsic stacking faults. The unstable TFE (*γ_u__tf_*) and TFE (*γ_tf_*) are defined at slip displacements of 1.5b and 2.0b, respectively. The *γ_u__tf_* represents the minimum energy required for twinning dislocations to nucleate in a perfect crystal. The evolutions of the *γ_i__sf_*, *γ_u__sf_*, *γ_tf_*, and *γ_utf_* as a function of W concentration are depicted in Figure 7b. It is noticeable that *γ_usf_*, *γ_isf_*, *γ_utf_*, and *γ_tf_* of Ir-W solid solutions decrease with an increasing W concentration.

Table 5 presents the calculated *γ_usf_*, *γ_isf_*, *γ_utf_*, and *γ_tf_* of pure Ir and Ir-W solid solution alloys, together with the known calculated and experimental values for comparison. The intrinsic SFE (*γ_isf_*) of pure Ir and Ir_31_W was 337.80 mJ/m^2^ and 280.97 mJ/m^2^, respectively, being in good agreement with the results of Xu et al. [65] (pure Ir: 349.00 mJ/m^2^; Ir_31_W: 277 mJ/m^2^). The calculated intrinsic SFE of pure Ir is about 20% lower than the experimental one (420 mJ/m^2^) [26]. The calculated *γ_usf_* and *γ_isf_* of Ir are in agreement with the previous research [66,67,68].

SFE studies on Ni, Co, and Mg suggest that the reduction of SFE results in an improvement of these materials’ toughness [28,29,30]. The reduction of unstable SFE is probably related to the lowering of the slip energy barrier [69]. Though the deformation mechanism of Ir is still not well understood, it is reasonable to predict that the reduction of unstable SFE will make slip easier, which results in the improvement in plastic deformation capacity of Ir.

The GSFE is also used to evaluate the twinning propensity. The high unstable TFE *γ_utf_* of Ir, about 880.83 mJ/m^2^, in the [11$\bar{2}$] direction hinders mechanical twinning formation. Tadmor et al. [67] proposed a theoretical model based on dislocation nucleation from crack tips, defining the twinning propensity parameter as a function of *γ_isf_*, *γ_usf_*, and *γ_utf_*,

(14) $$\tau_{a}=\left[1.136-0.151\frac{\gamma_{isf}}{\gamma_{usf}}\right]\sqrt{\frac{\gamma_{usf}}{\gamma_{utf}}}$$

A larger *τ_a_* indicates a higher propensity for twinning. Asaro et al. [70] proposed another twinning propensity parameter based on the heterogeneous nucleation mechanism of Shockley dislocations at grain boundaries,

(15) $$T=\sqrt{\frac{{3\;\gamma }_{usf}-{2\;\gamma }_{isf}}{\gamma_{utf}}}$$

A larger *T* value signifies easier twinning formation at grain boundaries. Cai et al. [66] proposed intrinsic twinning propensity *η* for FCC metals based on the homogeneous nucleation mechanism of Shockley dislocations,

(16) $$\eta =\frac{\gamma_{usf}-\gamma_{isf}}{\gamma_{utf}-\gamma_{isf}}$$

Table 6 presents the calculated results of the three twinning propensity parameters (τ*_a_*, *T*, and *η*) for Ir and Ir-W solid solutions. With increases in W content, the values of the three twinning criteria consistently increase, suggesting that the addition of W facilitates the heterogeneous nucleation of twins from crack tips and grain boundaries, as well as homogeneous nucleation within grains, thus enhancing the twinning propensity of Ir.

### 3.5. Electronic Structure Analysis

**Figure 8:**
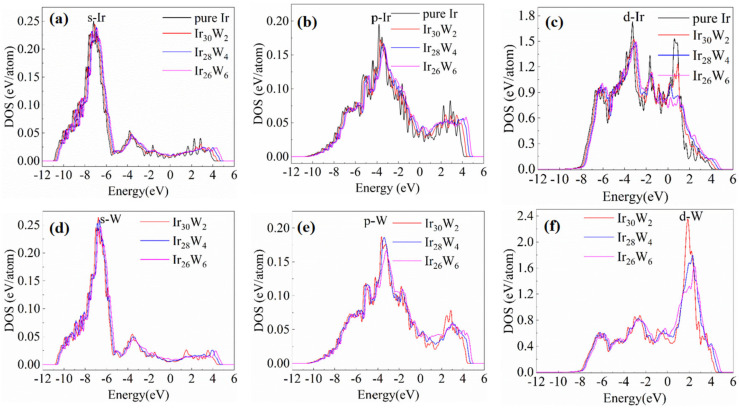
The s-, p-, d-orbital partial DOS of Ir atoms bonded with W of pure Ir, Ir_30_W_2_, Ir_28_W_4_, and Ir_26_W_6_ (**a**), (**b**), and (**c**), respectively, the s-, p-, d-orbital partial DOS of W sites of Ir_30_W_2_, Ir_28_W_4_, and Ir_26_W_6_ (**d**), (**e**), and (**f**), respectively.

The electronic density of states (DOS) of pure Ir and Ir-W solid solution alloys is presented in Figure 8. Panels (a), (b), and (c) show the s-, p-, d-orbital partial DOS for pure Ir and Ir atoms bonded with W in Ir_30_W_2_, Ir_28_W_4_, and Ir_26_W_6_. Panels (d), (e), and (f) are those at W sites in Ir_30_W_2_, Ir_28_W_4_, and Ir_26_W_6_. The Fermi level is at 0 eV.

Those Ir-Ir and Ir-W bonds exhibit metallic behavior owing to the absence of a bandgap of density of states near the Fermi level. The d-orbital DOSs of Ir and W are significantly higher than those of s- and p-orbitals, indicating that Ir-Ir and Ir-W bonds are mainly contributed by the d-orbital electrons. As the W content increases, all curves shift towards the positive direction, and the valleys in the d-DOS of Ir near −2.5 eV and −0.6 eV become shallower. The evolution of the valley depth partly reflects changes in the covalent properties of the metallic bonds. The covalent nature of Ir-Ir bonds is weakened with increasing W concentration.

**Figure 9:**
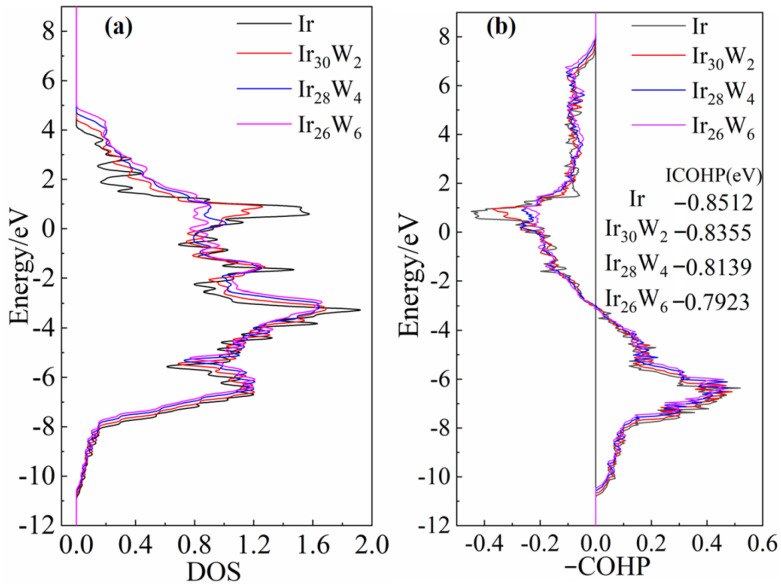
(**a**) The averaged total DOS of Ir and Ir atoms in pure Ir, Ir_30_W_2_, Ir_28_W_4_, and Ir_26_W_6_, (**b**) The projected crystal orbital Hamilton population (COHP) of Ir−Ir pairs in Ir, Ir_30_W_2_, Ir_28_W_4_, and Ir_26_W_6_.

Figure 9a presents the averaged total DOS of Ir and Ir atoms in Ir_30_W_2_, Ir_28_W_4_, and Ir_26_W_6_. As W content increases, the total DOS of Ir shifts toward the positive energy direction which suggests more overlap between bonding and antibonding states. Figure 9b presents the COHP curves of Ir-Ir atom pairs of Ir, Ir_30_W_2_, Ir_28_W_4_, and Ir_26_W_6_. The positive COHP (right) region of COHP plots is mainly contributed by bonding states, and the negative COHP (left) region is mainly contributed by the antibonding states. The integral COHP (ICOHP) value, with integrates COHP from the far negative to the Fermi level, is closely related to bond strength. The more negative the ICOHP value is, the stronger the bond is [36,37]. The ICOHP values for Ir-Ir bonds in pure Ir and Ir_30_W_2_, Ir_28_W_4_, and Ir_26_W_6_ are −0.8512 eV, −0.8355 eV, −0.8139 eV, and −0.7923 eV, respectively. ICOHP of pure Ir is the most negative one. This fact indicates the Ir-Ir bonds in pure Ir are stronger than those in the solid solutions. The absolute value of ICOHP decreases with increasing W content, which indicates that Ir-Ir bond strength is weakened.

**Figure 10:**
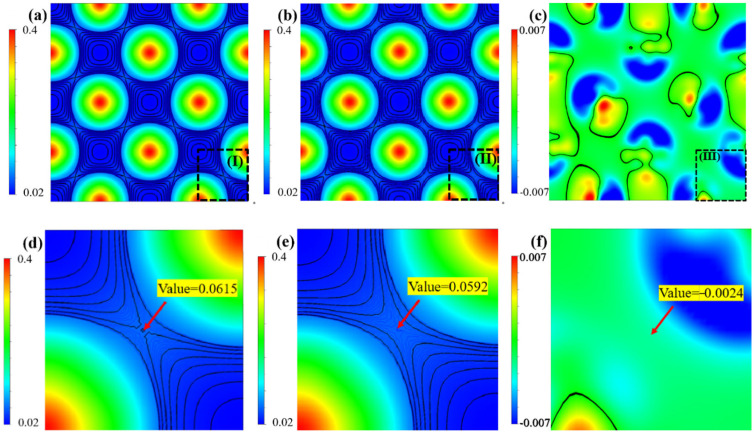
Charge density of pure Ir (**a**) and Ir_26_W_6_ (**b**) on the (010) plane; (**c**) depicts the charge density difference between Ir_26_W_6_ and Ir; (**d**–**f**) present the enlarged view of regions I, II, III in (**a**–**c**). The charge density contour lines for (**a**,**b**,**d**,**e**) are plotted from 0.02 to 0.4 eV/Å^3^ with a 0.0008 eV/Å^3^ intervals. The black lines in (**c**,**f**) are the zero-charge density contours.

Figure 10a,b show the calculated charge density distributions on the (010) plane for pure Ir and Ir_26_W_6_. The selected (010) plane contains only Ir atoms. The charge density contour lines for Figure 10a,b are plotted from 0.02 to 0.4 eV/Å^3^ with a 0.0008 eV/Å^3^ intervals. Figure 10d,e are enlarged views of regions (I, II) in Figure 10a,b. The charge densities between the neighboring Ir-Ir atoms in the middle region, shown in Figure 10d,e, are approximately 0.0615 eV/Å^3^ for pure Ir and 0.0592 eV/Å^3^ for Ir_26_W_6_, respectively. This fact indicates that fewer electrons are shared by neighboring Ir-Ir atomic pairs with W addition. The charge density of the Ir_26_W_6_, subtracting that of pure Ir, namely charge density difference, is shown in Figure 10c. Figure 10f is an enlarged view of region (III) in Figure 10c, and the black lines are the zero-charge density contours. It is noticeable that most regions between the neighboring Ir-Ir atomic pairs are green with negative values in Figure 10c,f. This is direct evidence of the reduction of shared electrons between neighboring Ir-Ir atomic pairs by W alloying.

## 4. Discussion

Ir, unlike other metallic elements of the FCC structure, exhibits inherent brittleness at RT. Kontsevoi et al. [71] suggested the brittleness of Ir is a result of the pseudo-covalent bond features of Ir-Ir bonds. Cawkwell et al. [72] suggested that there are two core screw dislocations in iridium, a glissile planar core and a metastable non-planar core. The athermal transformation between the two core structures leads to exceptionally high rates of cross-slip during plastic deformation, associated with an exponential increase in the dislocation density. Cawkwell et al. [72] reported that the mechanism of thermal cross-slip is because of the mixed metallic and covalent interatomic bonding. Kontsevoi et al. [71] suggested the effective way to increase plasticity of Ir-based alloy is to decrease the covalent contribution to chemical bonding by alloying methods.

The above DOS and COHP analyses evidence that the chemical bond of Ir-Ir is a combining of metallic and covalent natures. The charge density analyses evidence that the density of the shared electrons by neighboring Ir-Ir atoms decreases with increasing W concentration. The covalent nature of Ir-Ir bonds is gradually weakened. The reduction of ICOHP values with increasing W content also suggests that the Ir-Ir bond strength is weakened. This approach of studying alloying effects through electronic structure (e.g., charge density, bond characteristics) and mechanical property correlations is consistent with DFT studies on other refractory systems. For instance, Hu et al. [22] employed first-principles calculations to reveal how V addition modulates pseudo-energy gaps in NbMoTaW-based high-entropy alloys. It also shows how V addition affects interatomic interactions and mechanical properties.

Table 7 summarizes the evolution trends with increasing W concentration from 0 to 18.75 at % for Cauchy pressure, Pugh’s ratio, Poisson’s ratio, stacking fault energy, twinning propensity parameter, and ICOHP values. The above findings, the increasing of Cauchy pressure (*C*_12_−*C*_44_), bulk Pugh’s ratio (*B*/*G*), and Poisson’s ratio with increasing W concentration, suggest that the toughness of Ir-W solid solution alloys is improved. The addition of W results in a reduction of intrinsic SFE and unstable SFE of Ir. This evolution trend may promote the full dislocations to extended dislocations. This trend is similar to the findings in Co-based alloys reported by Tian et al. [29]. Alloying of Cr and W leads to a reduction of SFE and facilitates the dissociation of full dislocations into Shockley partials. These behaviors enhance ductility by hindering cross-slip and reducing creep rates. The reduction of unstable SFE also indicates that the energy barrier of slipping decreases [69]. With increasing the concentration of W, both the TFE (*γ_tf_*) and unstable TFE (*γ_utf_*) of Ir-W solid solutions decrease, resulting in increasing the twinning propensity parameters (τ*_a_*, *T*, and *η*), thus improving the twinning propensity of Ir.

Efforts to balance the strength and ductility of a material have been ongoing for centuries. Liu et al. [73] designed Ti–Zr–Nb–Ta and Ti–Zr–Nb–Mo refractory HEAs balancing strength and ductility through tailoring valence electron concentration by changing the alloying composition. Chen et al. [74] investigated a CoCuFeNiPd HEA, which undergoes short-range ordering (SRO). The SRO leads to a pseudo-composite microstructure, which surprisingly enhances both the ultimate strength and ductility. Face-center-cubic-preferred clusters enhance strength, while body-center-cubic-preferred clusters improve ductility. Qi et al. [75] developed single-phase ordered B_2_ aluminum-enriched refractory HEAs demonstrating high strength and ductility. It is reported that valence electron count (VEC) defines domains for alloy ductility and brittleness. Strategically avoiding the VEC valley is advised in designing future ductile alloys.

Pei et al. [76] designed HEAs with enhanced strength and ductility. They highlighted that negative SFE, combined with a *k* parameter (ratio of short-ranged interactions between closed-packed planes) with |*k*| close to 1/2, promotes the formation of various nanoscale close-packed structures (such as twins, stacking faults, and nano-sized HCP domains). These structures balance strength and ductility by tailoring SFE and atomic interactions. Ren et al. [77] reported that the alternating FCC and BCC nanolamellae in AlCoCrFeNi eutectic HEAs synergistically enhanced strength and ductility. The present research established that it is theoretically practicable to improve the toughness of Ir by alloying methods with tailoring valence electron concentration to decrease the covalent contribution to chemical bonding. Alloying Ir with W results in the weakening of Ir-Ir covalent properties and the reduction of SFE. The toughening mechanism is consistent with these advanced alloy design concepts.

Shang et al. [28] stated that SFE in Ni-based alloys decreases with increasing temperature, which may further modulate deformation mechanisms under service conditions. Yang et al. [78] reported that the ductile–brittle transition temperature of Ir is in the range of 700–800 °C. They attributed low-temperature brittleness (25–700 °C) to high SFE. High SFE raises the activation barrier for dislocation slip, which hinders screw dislocation migration and causes dense dislocation tangles. The 0 K simulation results elucidate the intrinsic properties for the enhancement of Ir toughness by W alloying (SFE and electronic properties). Thermal effects at finite temperatures may improve the toughness of Ir alloys. These effects include lattice parameter changes caused by thermal expansion and elastic modulus softening due to phonons. The evolution of toughness as a function of temperature may also be affected by W. For example, W may influence toughness and the brittle transition temperature. The evolution of elastic properties and SFE of Ir and Ir-W solid solution alloys as a function of temperature will be investigated in future work.

## 5. Conclusions

In the present research, first-principles calculations were carried out, focusing on the effect of W on the elastic properties, GSFE, electronic structures, and Ir-Ir chemical bonding properties of Ir-W solid solution alloys. The calculated lattice constants, elastic constants, and elastic modulus of Ir, Ir_3_W (ε′), and IrW (ε′′) are in good agreement with the reported theory and experimental values. The calculated GSFEs of Ir are in good agreement with the reported values. The evolution of lattice constants, formation energies, elastic constants, elastic modulus, GSFE, and electronic structures of Ir-W solid solutions alloys as a function of W concentration was addressed and compared. The main conclusions are summarized as follows.

(1) Cauchy pressure (C12−C44) increases from −22 GPa to 5 GPa, and Pugh ratio (B/G) increases from 1.60 to 1.72 with increasing W content from 0 to 18.75 at %. These facts suggest that the toughness of Ir-W alloys is improved.
(2) The intrinsic SFE and unstable SFE of Ir alloy decrease with increasing W concentration. It is reasonable to predict that the reduction of SFE accompanies the lowering of the slipping barrier, which is responsible for the improvement of the material’s plastic deformation capability.
(3) The Ir-Ir bonds exhibit both metallic and covalent natures. The covalent nature of Ir-Ir bonds is weakened with the increasing of W concentration, evidenced by the growing ICOHP values from −0.8512 eV to −0.7923 eV and the reduction of common shared electrons between neighboring Ir-Ir atoms.

The weakening covalent character of Ir-Ir bonds through W alloying results in a decrease in stacking fault energy and lowering the energy barrier for sliding. It is a feasible approach to improve the toughness of Ir and mitigate its inherent brittleness by alloying methods. Further research, including both theoretical calculations and experimental studies, is strongly desired to design and develop high-temperature Ir-based alloys with higher toughness and strength.

## Figures and Tables

**Table 1:** Key DFT calculation parameters used in the modeling.

	Pure Ir	Ir_32−x_W_x_	Ir_4_W	Ir_3_W	IrW	GSFE
plane-wave basis cutoff energy	550 eV					550 eV
convergence criterion for electronic steps	1 × 10^−6^ eV/atom					1 × 10^−4^ eV/atom
convergence criterion for ionic steps	0.005 eV/Å					0.01 eV/Å
magnetic moment	not addressed					not addressed
k-point mesh	8 × 8 × 8		24 × 24 × 1	12 × 11 × 11	11 × 18 × 10	4 × 4 × 1

**Table 2:** Experimental (Exp.) and calculational (Cal.) lattice constant of pure Ir, Ir_4_W, Ir_3_W, and IrW and supercell length of Ir-W solid solution 2 ×  2 × 2 supercells (a, b, c), and mean bond length of Ir-Ir and Ir-W.

Element	Crystal Type	Lattice Constant or Supercell Length (Å)			Mean Bond Lengths (Å)		Reference
		a	b	c	Ir-Ir	Ir-W	
Ir	Cubic	3.873			2.738		This work
	Cubic	3.902					Cal. [12]
	Cubic	3.876					Cal. [13]
	Cubic	3.874					Cal. [23]
	Cubic	3.819					Exp. [51]
	Cubic	3.840					Exp. [52]
	Cubic	3.903					Cal. [53]
	Cubic	3.912					Cal. [54]
Ir_31_W_1_	Cubic	7.751			2.741	2.728	This work
	Cubic	7.768					Cal. [13]
Ir_30_W_2_	Cubic	7.758			2.745	2.719	This work
Ir_29_W_3_	Cubic	7.765			2.749	2.720	This work
Ir_28_W_4_	Cubic	7.773			2.755	2.721	This work
Ir_27_W_5_	Cubic	7.779			2.757	2.725	This work
Ir_26_W_6_	Cubic	7.788			2.762	2.724	This work
Ir_4_W	Trigonal	2.751	2.751	33.850			This work
	Trigonal	2.760	2.760	33.930			[44]
Ir_3_W (ε′)	Hexagonal	5.540	5.540	4.421			This work
	Hexagonal	5.551	5.551	4.390			Cal. [46]
	Hexagonal	5.515	5.515	4.409			Exp. [55]
IrW (ε′′)	Orthorhombic	4.473	2.779	4.848			This work
	Orthorhombic	4.486	2.788	4.866			Cal. [46]
	Orthorhombic	4.469	2.773	4.825			Exp. [55]

**Table 3:** Elastic constants of IrW alloy (GPa).

	C_11_	C_12_	C_13_	C_14_	C_22_	C_23_	C_33_	C_44_	C_55_	C_66_	Reference
Ir	581	235						257			This work
	657	280						281			Cal. [12]
	642	269						282			Cal. [13]
	594	236						248			Cal. [23]
	597	247						262			Cal. [53]
	595	239						256			Cal. [54]
	583	229						258			Cal. [57]
	573	223						245			Cal. [58]
Ir_31_W_1_	578	235						252			This work
	628	269						271			Cal. [13]
Ir_30_W_2_	571	235						246			This work
Ir_29_W_3_	563	235						242			This work
Ir_28_W_4_	555	236						238			This work
Ir_27_W_5_	552	235						237			This work
Ir_26_W_6_	542	232						227			This work
Ir_4_W	613	224	173	35			662	172		194	This work
Ir_3_W	636	205	214				684	175		216	This work
	622	200	214				661	171		211	Exp. [55]
IrW	635	228	196		592	174	662	155	184	173	This work
	618	223	194		583	171	632	158	186	173	Exp. [55]

**Table 4:** Elastic modulus (*B*, *G*, and *E)*, Pugh’s ratio (*B*/*G*), and Poisson’s ratio (ν) of Ir-W alloy (GPa).

Element	*B*	*G*	*E*	*B*/*G*	ν	Reference
Ir	350.18	219.00	543.66	1.60	0.241	This work
	360.00	220.00		1.64		Exp. [5]
	406.49	238.50	598.45	1.70	0.254	Cal. [12]
	393.00	239.00	596.00	1.65	0.248	Cal. [13]
	355.00	220.00	547.00	1.61	0.240	Cal. [23]
	364.00	223.00	556.00	1.63	0.246	Cal. [53]
	358.00	221.00	550.00	1.62	0.244	Cal. [54]
	346.90	221.84	548.61	1.56	0.236	Cal. [57]
	339.00	212.00	526.00	1.60	0.241	Cal. [58]
	370.70	209.90		1.77		Exp. [61]
Ir_31_W_1_	349.34	216.18	537.63	1.62	0.244	This work
	393.00	236.00	589.00	1.67	0.250	Cal. [13]
Ir_30_W_2_	346.80	211.22	526.73	1.62	0.247	This work
Ir_29_W_3_	344.16	207.03	517.34	1.66	0.249	This work
Ir_28_W_4_	342.04	202.80	507.99	1.69	0.252	This work
Ir_27_W_5_	340.55	201.95	505.86	1.69	0.252	This work
Ir_26_W_6_	335.51	194.91	489.87	1.72	0.257	This work
Ir_4_W	336.57	191.05	481.96	1.76	0.261	This work
Ir_3_W	357.65	200.06	505.87	1.79	0.264	This work
	351.00	194.00		1.81	0.270	Exp. [55]
IrW	342.38	186.57	473.67	1.84	0.269	This work
	334.00	184.00			0.270	Exp. [55]

**Table 5:** The *γ_usf_*, *γ_is_f*, *γ_ut_f*, and *γ_tf_* of pure Ir and Ir-W solid solutions.

	*γ_usf_* (mJ/m^2^)	*γ_isf_* (mJ/m^2^)	*γ_utf_* (mJ/m^2^)	*γ_tf_* (mJ/m^2^)	Reference
Pure Ir	636.90	337.80	880.83	354.56	This work
		420.00			Exp. [26]
		349.00			Cal. [65]
	740.90	282.60			Cal. [66]
	679.00	305.00			Cal. [67]
	625.00	334.00			Cal. [68]
Ir_31_W	618.25	280.97	819.7	244.84	This Work
		277.00			Cal. [65]
Ir_30_W_2_	589.90	188.65	810.03	196.28	This Work
Ir_29_W_3_	620.19	158.17	797.53	185.6	This Work
Ir_28_W_4_	523.39	37.37	680.74	77.72	This Work
Ir_27_W_5_	603.51	86.29	654.78	64.17	This Work
Ir_26_W_6_	547.39	21.16	456.3	35.64	This Work

**Table 6:** Twinning propensity parameter for Ir and Ir-W solid solutions.

	$\tau_{a}$	*T*	η
Pure Ir	0.898	1.184	0.551
Ir_31_W	0.935	1.270	0.645
Ir_30_W_2_	0.928	1.311	0.646
Ir_29_W_3_	0.968	1.391	0.723
Ir_28_W_4_	0.987	1.482	0.755
Ir_27_W_5_	1.070	1.582	0.910
Ir_26_W_6_	1.238	1.874	1.209

**Table 7:** Summary of the evolution trend with increasing W concentration from 0 to 18.75 at % for Cauchy pressure, Pugh’s ratio, Poisson’s ratio, stacking fault energy, twinning propensity parameter, and ICOHP values.

Properties of Ir-W Solid Solution	Evolution Trend with Increasing W Concentration from 0 to 18.75%
Cauchy pressure (*C*_12_–*C*_44_)	become larger
Pugh’s ratio (*B*/*G*)	become larger
Poisson’s ratio (ν)	become larger
intrinsic stacking fault energy (*γ_isf_*)	decrease
unstable stacking fault energy (*γ_usf_*)	decrease
twinning fault energy (*γ_tf_*)	decrease
unstable twinning fault energy (*γ_utf_*)	decrease
twinning propensity parameter ($\tau_{a}$, *T*, *η*)	increase
shared electrons by neighboring Ir-Ir atoms	decrease
ICOHP values	reduction
covalent properties of Ir-Ir bond	weakened
toughness	improved

## Data Availability

The original contributions presented in this study are included in the article. Further inquiries can be directed to the corresponding authors.
